# Neuroprotective Effect of Maresin-1 in Rotenone-Induced Parkinson’s Disease in Rats: The Putative Role of the JAK/STAT Pathway

**DOI:** 10.1007/s11064-024-04282-x

**Published:** 2024-11-22

**Authors:** Suzan A. Khodir, Eman M. Sweed, Manar A. Faried, Doaa M. Abo Elkhair, Marwa M. Khalil, Khaled Hatem Afifi, Dalia Fathy El Agamy

**Affiliations:** 1 Medical Physiology Department, Faculty of Medicine, Menoufia University, Menoufia, 32511 Egypt; 2 Medical Physiology Department, Menoufia National University, Menoufia, Egypt; 3 Clinical Pharmacology Department, Faculty of Medicine, Menoufia University, Menoufia, 32511 Egypt; 4 Quality Assurance Center, Menoufia National University, Menoufia, Egypt; 5 Anatomy and Embryology Department, Faculty of Medicine, Menoufia University, Menoufia, 32511 Egypt; 6 Medical biochemistry and molecular biology Department, Faculty of Medicine, Menoufia University, Menoufia, 32511 Egypt; 7 Medical biochemistry and molecular biology Department, Menoufia National University, Menoufia, Egypt; 8 Neurology Department, Faculty of Medicine, Menoufia University, Menoufia, 32511 Egypt

**Keywords:** Acetylcholine, Dopamine, JAK1, Maresin-1, NF-kB, Parkinson’s disease, STAT3

## Abstract

Exposure to rotenone results in similar pathophysiological features as Parkinson’s disease. Inflammation and oxidative stress are essential to PD pathogenesis. Maresin-1 has potent anti-inflammatory properties and promotes the regression of inflammation function. The current study aimed to evaluate the protective effects of Maresin-1 (MaR1) in rotenone (ROT)-induced PD and whether this protective role is associated with the initiation of the Janus kinase (JAK)-signal transducers and activator of transcription (STAT) signaling pathway. Thirty male Wister rats were classified into control, ROT-treated, and ROT + MaR1-treated groups. Rats underwent rotarod, open field, grip strength, and stepping tests as part of their motor behavioral evaluation. Serum glial cell-derived neurotrophic factor (GDNF) and striatal dopamine, acetylcholine, malondialdehyde (MDA), reduced glutathione (GSH), TNF-α, IL-6, and IL-1β were evaluated. Expression of JAK1 and STAT3 genes was assessed in striatum. Then, the tissue was subjected to histological and immunohistochemical evaluation for caspase-3, GFAP, and NF-kB. The administrated group with rotenone showed significant motor behavioral impairment. This was accompanied by reduced levels of GDNF and dopamine and increased levels of acetylcholine, as well as augmented oxidative stress and inflammatory biomarkers and reduced antioxidant activity. Inflammatory pathways (JAK1/STAT3, caspase-3, and NF-kB) were upregulated. Histopathological changes and upregulation in GFAP immunopositive reaction were observed. Remarkably, MaR1 treatment effectively alleviated behavior, histopathological changes, and biochemical alterations induced by ROT. MaR1 exerts protective effects against ROT-induced PD by its anti-inflammatory, antiapoptotic, and antioxidant properties. MaR1 mechanisms of action may involve modulation of pathways such as JAK/STAT.

## Introduction

Parkinson’s disease (PD) is a neurodegenerative disease that leads to debilitating motor function impairment. The primary feature of PD is the progressive decline of substantia nigra dopamine-secreting neurons ensuing in dopamine depletion in the striatum, which is the main substantia nigra projection area. This results in an unbalance between brain acetylcholine and dopamine levels causing PD symptoms. PD is distinguished by clinical symptoms including bradykinesia, resting tremors, gait impairment, and sensory-motor integration deficits [1]. The etiology of PD remains mostly unexplained. Nonetheless, recent experimental and epidemiological research has reignited interest in the theory that environmental factors are crucial to the pathophysiology of PD. On the contrary, genetic factors account for a small fraction of PD cases [2].

Rotenone is a well-known pesticide. Chronic rotenone administration results in PD-like biochemical and behavioral changes in rats, and compared to other models, the rotenone rat model of PD would have certain advantages for experimental investigations as it more closely resembles the long-term course of PD as seen in patients and it can lead to selective vulnerability for nigrostriatal degeneration [3]. Chronic systemic rotenone exposure produces nigrostriatal damage and destroys dopaminergic neurons resembling the human clinical situation. It causes oxidative damage, α-synuclein buildup, cell death, mitochondrial degradation, and altered calcium signaling by mitochondrial complex I electron-transport chain suppression [4, 5]. Inflammation and oxidative stress are vital to PD pathogenesis. The Janus kinase (JAK)-signal transducers and activator of transcription (STAT) signaling pathway and other transcription factors are increased in response to neuroinflammation, which triggers microglial activation and results in PD via dopaminergic neuron autophagy [6, 7]. The binding of a ligand to its appropriate receptor successively activates receptor-associated JAKs and STATs leading to rapid signaling from the cell surface to the nucleus, driving biological responses to cytokines [8]. The JAK-STAT pathway inhibition stops neuroinflammation and neurodegeneration [9].

A variety of endogenous anti-inflammatory mediators, known as specialized pro-resolving mediators or SPMs, participate in the dynamic and well-coordinated process of inflammation resolution [10]. In immune cells, SPMs are naturally produced from omega-3 and omega-6 polyunsaturated fatty acids by a group of enzymes known as lipoxygenases, specifically 12- and 15-LOX [11]. Maresin-1 (MaR1), a naturally omega-3 fatty acids-derived lipid mediator, is synthesized and released by macrophages. SPM exogenous treatment could significantly reduce symptoms in several inflammatory-related diseases [12]. Thus, the present study aimed to investigate the potential protective effects of MaR1 in this experimental model of PD and whether these effects are associated with the JAK-STAT signaling activation.

## Materials and methods

### Experimental Animals

#### Sample Size Calculation

Thirty male Wistar rats, eight to twelve weeks old, weighing 200–220 g, were utilized. The sample size was calculated using the Resource Equation Approach to estimate the maximum number while taking care to avoid the dropout rate. An 80% study power and a 95% confidence interval were utilized for calculating the sample size.

Five animals per cage were kept in a particular pathogen-free state at a temperature of 24 ± 3 °C with a 12-hour light/dark cycle with unrestricted availability of food and water. The Menoufia University Institute, Faculty of Medicine’s Animal Care and Use Committee (approval no. 3/2024 BIO 13-2) authorized all animal experimental procedures utilized in the present work. The animal study complied with the guidelines for Reporting In Vivo Experiments (ARRIVE) in Animal Research.

##### Group 1 (Control Group)

The vehicle-treated group received rotenone vehicle injection subcutaneously (s.c.), 25 µL/kg dimethyl sulfoxide (DMSO) (Fisher Scientific, Loughborough, UK) and 1.25 mL/kg sterile sunflower oil once a day for 5 weeks, and intraperitoneal (i.p.) DMSO injection for 8 weeks.

##### Group 2 (rotenone-induced PD (ROT) Group)

2 mg/kg rotenone (R8875-1G, Sigma Aldrich, Steinheim, Germany) was injected s.c. once a day for 5 weeks [13, 14]. Rotenone powder was suspended in 25 µL/kg DMSO and 1.25 mL/kg sterile sunflower oil. To guarantee uniform distribution, the suspension was well mixed right before injection. It was freshly prepared daily.

##### Group 3 (rotenone-induced PD Maresin-1-treated (ROT + MaR1) Group)

Rats received rotenone as in group 2 + daily i.p. 4 ng/g per BW of MaR1 (Cayman Chemical, Ann Arbor, MI, USA), 50 mg of Mar1 was dissolved in 1 ml of DMSO for 8 weeks [15–17]. The MaR1 dosage was selected to exceed the levels associated with approved MaR1 bioactivity in experimental disease models, as well as this dosage is adequate to exert bioactivity in the central nervous system in an intact and closed blood-brain barrier [15].

Rats received MaR1 once daily for 3 weeks before rotenone injection and continued together with rotenone for the next 5 weeks with 3 h intervals between them. 

### Motor behavioral tests

The following motor behavioral tests were evaluated at the end of the experiment, just before the collection of blood samples and rat scarification, for motor function evaluation. These investigations were all carried out in a quiet environment at ambient temperature and without any outside interference.

#### Open Field Test

Rats’ innate locomotor activity and exploratory tendencies were observed. The apparatus was built from a square wooden box measuring 80 by 80 by 40 centimeters. The floor was divided into 16 (4 × 4) squares by white lines. Rats were put in the open field’s center. Their movements were captured on camera for 5 min. Latency time (the time it took to depart from the starting point), ambulation frequency (the number of squares that the rats crossed), and rearing frequency (how often the rat stands on its hind legs) were evaluated. To get rid of any rodent odors, the box was thoroughly cleaned with 10% alcohol and allowed to dry before the test was carried out in a calm area with dim white lighting [18, 19].

#### Grip Strength Test

A digital grip strength meter (GSM Grip Strength Meter 47200; UGO BASILE S.R.L., Italy) was utilized to evaluate the grip strength of a rat forelimb. Rats were held gently from their tails onto the grid, allowing them to hold the T-shaped bar exclusively with their front paws. The grip strength was monitored in gram force (gf) as the rat’s documented peak power was on the GSM before departing the bar. Three testing readings were utilized to calculate the average value for each rat [20].

#### Rotarod Test

Rats’ motor coordination was evaluated using the rotarod test. A rotating rod RR02 (58 cm length, 31 cm diameter, and 51 cm height) rotated at a fixed speed of 26 rpm with a rotator diameter of 6 cm, lane width of 8.7 cm, falling height of 32 cm, and lane separator diameter of 31 cm (Orchid Scientific & Innovative, Ambad, Nashik, India). Before the experiment, for three days, each rat completed three trials lasting five minutes apiece, with a five-minute rest in between to allow the rats to get used to using the rotarod. Time spent on rotarod was recorded [19, 21].

#### Stepping Test

The stepping test is designed to detect motor starting impairments in the forelimbs, similar to limb akinesia and gait disorders in PD patients. The rat’s hindquarters were securely suspended as it used its forelimbs to sustain its weight. The rat then made three rearward movements along the table in five seconds (0.9 m). The total number of adjusting steps noted in the three tests was added up to establish the session’s overall score [22].

### Collection of Blood Samples and Separation of Serum

At the trial conclusion, following a neurological evaluation, using tiny, heparinized capillary tubes implanted into the medial epicanthus of their eyes, fasting blood samples were obtained from the retro-orbital venous plexus. After allowing 2 milliliters of blood to clot, the sample was spun for 15 min at 3000 revolutions per minute in a graduated centrifuge tube. A dry, clean tube was utilized for collecting the supernatant serum for glial cell-derived neurotrophic factor analysis (GDNF) using rat GDNF enzyme-linked immunosorbent assay (ELISA) kit (Cat.: ab213901, Abcam, Cambridge, UK) according to the manufacturer instructions.

Rats were then decapitated and sacrificed. After being gently removed, the brains were rinsed in ice-cold saline. An ice-cold glass dish was utilized to dissect each brain. The basal ganglia of each brain’s left hemisphere were separated, weighed, and divided into two parts; the first half stored at -80oC for rt- PCR and the other half homogenized using glass rods. Homogenization was performed at 10% (w/v) in a phosphate-buffered solution (pH = 7). The homogenate was centrifuged at 3000 × g for 10 min. The supernatants were stored at -80 °C until the striatal tyrosine Hydroxylase (TH) (Cat.: MBS701326, MyBioSource, Sandiego, CA, USA), striatal dopamine (Cat.: 201-11-0220, Sunred CO., Shanghai, China) and acetylcholine (Cat.: 201-11-0723, Sunred CO., Shanghai, China) were measured using rat ELISA kits according to the manufacturer instructions. Striatal malondialdehyde (MDA) and reduced glutathione (GSH) were measured spectrophotometrically using available commercial kits (Biodiagnostic CO., Dokki, Giza, Egypt). Striatal TNF-α (Cat.: MBS2507393, MyBioSource, Sandiego, CA, USA), IL-6 (Cat.: MBS269892, MyBioSource, Sandiego, CA, USA), and IL-1β were measured using ELISA Kit (Cat.: ab100768, Abcam, Cambridge, UK).

### RT-PCR Gene Profiling of JAK1 and STAT3

The Trizol technique (Invitrogen) was utilized for isolating the total RNA from tissues and cells. To make cDNA, two micrograms of the total RNA was utilized. In a final volume of 20 µL, each PCR reaction was carried out with 10 µL of SYBR green (2x quantiTect PCR master mix), 3 µL of cDNA, 1 µL of forward primer, 1 µL of reverse primer, and 5 µL of RNase free water. The following conditions were met during the amplification: 94 °C for two minutes, followed by 35 cycles of denaturation (30 s at 94 °C), annealing (30 s at 58 °C), and extension (45 s at 72 °C). A final 10-minute extension at 72 °C came next. The following primers were utilized to evaluate gene expression for JAK1: forward primer; GAGGAATGTACTGGGCGTCT and reverse primer; TGCAGCCGGAGAGACATTTT; for STAT3: forward primer; ATGTCTCAAGATGGCGGAGC and reverse primer; GACCGACAGCCAGTCAAAGA.

### Histopathological Analysis

After dissection of the brains, their right hemispheres were fixed in 10% neutral buffered formalin. Paraffin Sect. (5-µm thick) were prepared and subjected for histological and immunohistochemical studies. The hematoxylin and eosin (H&E) stain was applied to substantia nigra pars compacta (SNc) sections. Paraffin sections were first deparaffinized and then rehydrated in preparation for immunohistochemical staining. Sections were incubated in a blocking solution (10% normal goat serum). Following that, the sections were treated with the primary antibodies against glial fibrillary acidic protein (GFAP) [mouse monoclonal, Lab vision MS-1376-R7, 1:300] and anti-caspase-3 (rabbit monoclonal, Abcam ab184787, 1:1000) and nuclear factor kappa *B (*NF-kB) p65, or anti-nuclear factor kappa beta [polyclonal rabbit, ab16502, 1:100]. Thereafter, after rinsing each section with PBS, the secondary biotinylated antibody was incubated. 3,3-diaminobenzoic acid (DAB) was utilized for visualizing the binding of secondary antibodies. Afterward, two drops of hematoxylin were used to counterstain the slides.

From each animal, five non-overlapping fields (x400) in each section were randomly captured using a Leica DML B2/11,888,111 microscope fitted with a Leica DFC450 camera for quantitative evaluation. The ImageJ software version K1.45 was utilized to determine the parameters under examination. The percentage of deteriorated neurons within the SNc was evaluated for sections labeled with H&E. The area percentage of GFAP, caspase-3, and NF-kB immunoreaction was computed for immunohistochemical quantitative assessment. The immunoreactive area and total area were measured using threshold feature in image J. The sum of the immunoreactive areas was divided by that of the total area and this quotient was multiplied by 100 to yield the %area of the positive immunoreaction.

### Statistical Analysis

Kolmogorov-Smirnov was utilized to assess the normal distribution of data. The data is presented as the mean ± standard deviation (SD) if normally distributed. The experiments were analyzed using one-way ANOVA and the Tukey post hoc test. All statistical analyses were conducted using SPSS (IBM Inc., Armonk, NY, USA) version 27 and GraphPad Prism 8 software (GraphPad Software Inc., La Jolla, CA, USA). It is considered statistically significant when *P* < 0.05.

## Results

### Motor behavioral results

#### Open field test results

**Fig. 1:**
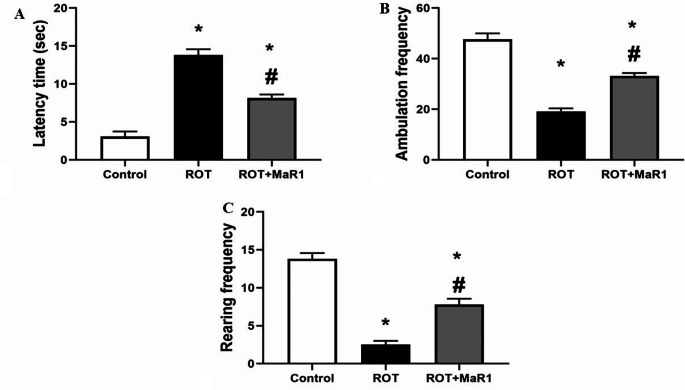
Maresin-1 impact on latency time (sec), ambulation frequency, and rearing frequency in rotenone-induced Parkinson’s disease. Data represented as mean ± SD (*n* = 10) and group comparisons were made utilizing the ANOVA test. (**a**) Latency time, (**b**) ambulation frequency, and (**c**) rearing frequency. ROT (rotenone-induced Parkinson’s disease) and ROT + MaR1 (rotenone-induced Parkinson’s disease treated with Maresin-1). *p < 0.05 is significant when compared to the control, and # *p* < 0.05 is significant when linked to the ROT group

Regarding the open field parameter, Comparing the ROT group to the control group, the ROT group had a significantly longer latency time and fewer ambulation and rearing episodes (13.83 ± 0.75 s vs. 3.08 ± 0.66 s, 19.16 ± 1.16 vs. 47.66 ± 2.33, and 2.5 ± 0.5 vs. 13.83 ± 0.75, respectively; *P* ≤ 0.05). However, the ROT + MaR1 group showed a significant reduction in latency time and significant elevation in ambulation and rearing frequency in comparison with the ROT group (8.16 ± 4.56 s, 33.16 ± 1.16, and 7.8 ± 0.75, respectively; *P* ≤ 0.05) (Fig. 1).

#### Grip strength test results

**Fig. 2:**
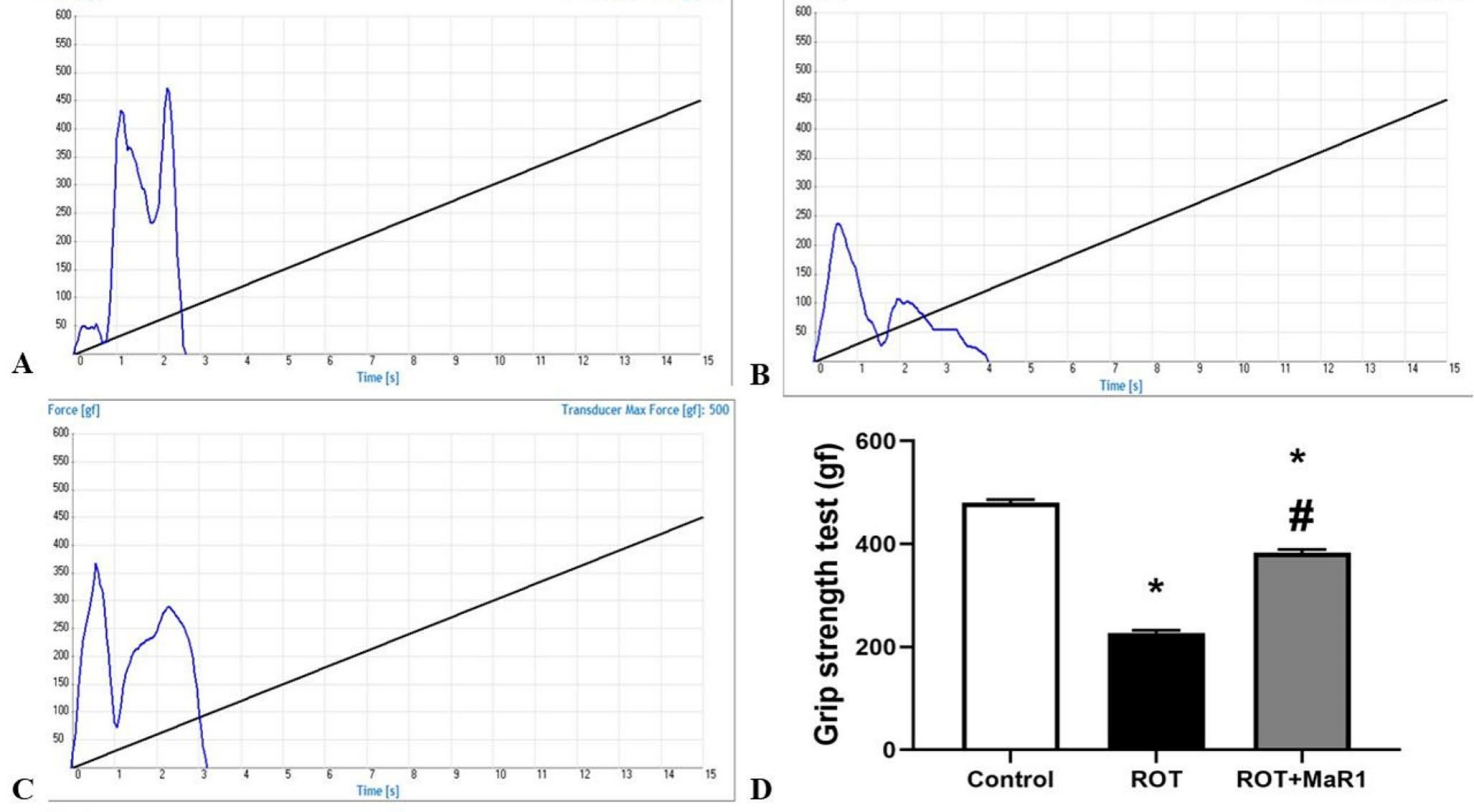
Maresin-1 impact on grip strength test in rotenone-induced Parkinson’s disease. Data represented as mean ± SD (*n* = 10) and group comparisons were made utilizing the ANOVA test. (**a**) Control group curve of grip strength test by a digital grip strength meter, (**b**) ROT group curve of grip strength test by a digital grip strength meter, (**c**) ROT + MaR1 group curve of grip strength test by a digital grip strength meter, and (**d**) grip strength test (gf). ROT (rotenone-induced Parkinson’s disease) and ROT + MaR1 (rotenone-induced Parkinson’s disease treated with Maresin-1). *p < 0.05 is significant when compared to the control, and # *p* < 0.05 is significant when linked to the ROT group

The ROT group exhibited a significant decline in grip strength in comparison with the control group (227.2 ± 5.11 vs. 480.0 ± 6.26 gf, respectively; *P* ≤ 0.05). However, ROT + MaR1 group showed a significant elevation in grip strength in comparison with the ROT group (383.3 ± 5.82 gf, *P* ≤ 0.05), while it is still significantly lower than the control group (Fig. 2).

#### Rotarod and stepping test results

**Fig. 3:**
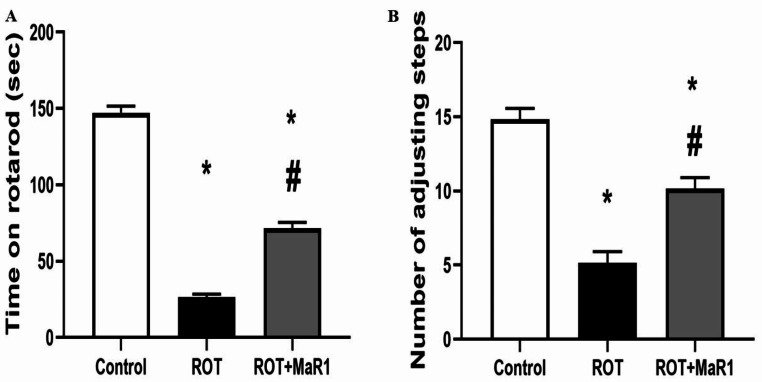
Maresin-1 impact on rotarod test (Sec) (**a**) and stepping test (steps) (**b**) in rotenone-induced Parkinson’s disease. Data represented as mean ± SD (*n* = 10) and group comparisons were made utilizing the ANOVA test. ROT (rotenone-induced Parkinson’s disease) and ROT + MaR1 (rotenone-induced Parkinson’s disease treated with Maresin-1). *p < 0.05 is significant when compared to the control, and # *p* < 0.05 is significant when linked to the ROT group

The ROT group exhibited a substantial decline in time on the rotarod and the number of adjusting steps in stepping tests compared to the control group (26.33 ± 2.06 vs. 147.2 ± 4.35 s and 5.16 ± 0.75 vs. 14.83 ± 0.75 steps, respectively; *P* ≤ 0.05). However, the ROT + MaR1 group showed a significant increase in time on the rotarod and in the number of adjusting steps compared to the ROT group (71.67 ± 3.83 s and 10.16 ± 0.75 steps, respectively; *P* ≤ 0.05), although these values remained substantially lower than those of the control group. (Fig. 3).

### Biochemical results

#### Serum GDNF, striatal TH, dopamine and acetylcholine results 

**Fig. 4:**
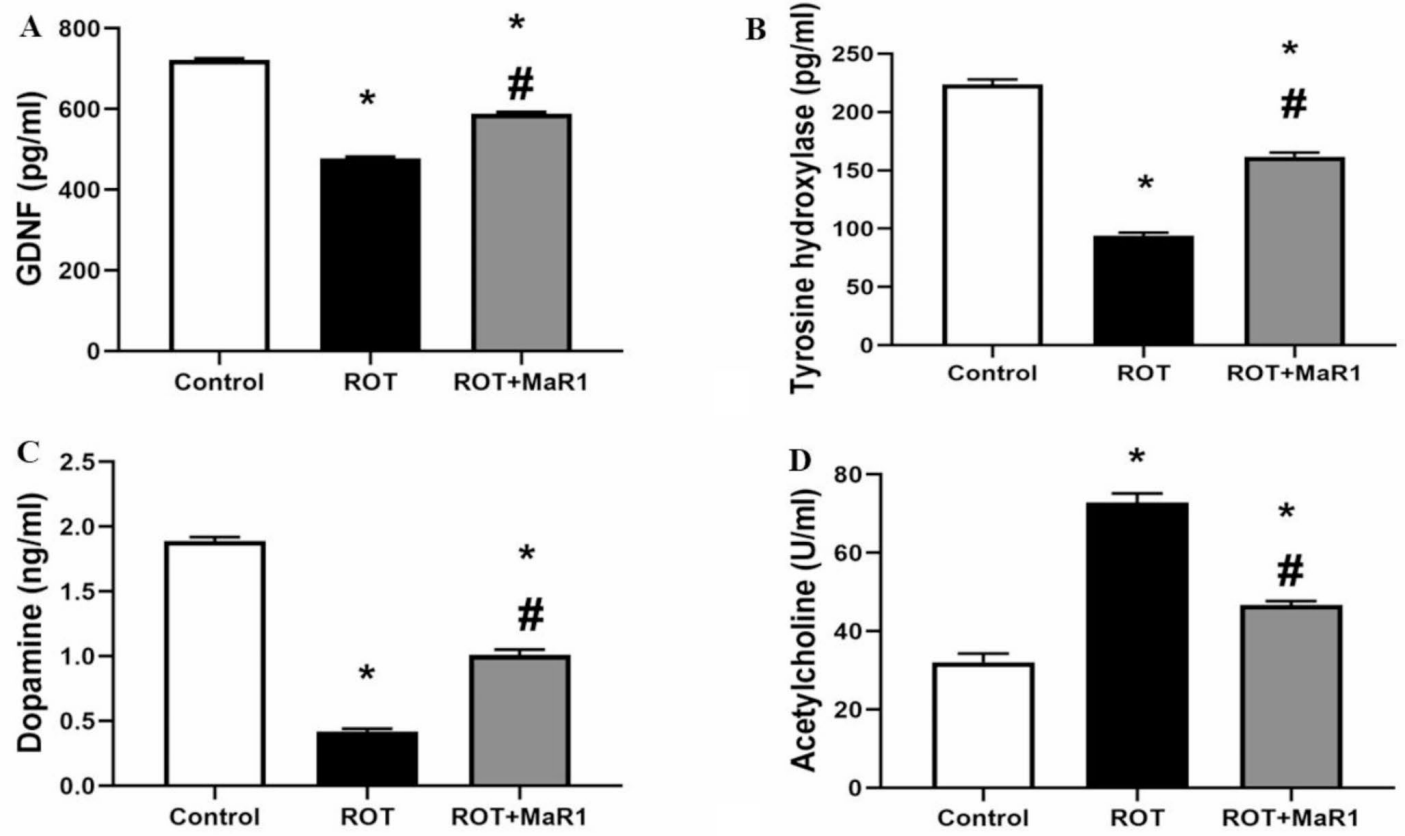
Maresin-1 impact on serum glial cell-derived neurotrophic factor (GDNF) (pg/ml) (**a**), Tyrosine hydroxylase (pg/ml) (**b**), striatal dopamine (ng/ml) (**c**), and striatal acetylcholine (U/ml) (d) in rotenone-induced Parkinson’s disease. Data represented as mean ± SD (*n* = 10) and group comparisons were made utilizing the ANOVA test. ROT (rotenone-induced Parkinson’s disease) and ROT + MaR1 (rotenone-induced Parkinson’s disease treated with Maresin-1). *p < 0.05 is significant when compared to the control, and # *p* < 0.05 is significant when linked to the ROT group

The ROT group showed a significant decline in the level of serum GDNF, striatal TH, and striatal dopamine in comparison with the control group (477.66 ± 4.5, 94 ± 2.53 pg/ml, 0.42 ± 0.02 ng/ml vs. 721.66 ± 3.77, 223.67 ± 4.37 pg/ml, and 1.89 ± 0.03 ng/ml; *P* ≤ 0.05). ROT + MaR1 group exhibited a significant elevation in serum GDNF, striatal TH, and striatal dopamine levels in comparison with the ROT group (588.16 ± 4.75, 161.67 ± 3.56 pg/ml, and 1.01 ± 0.04 ng/ml; *P* ≤ 0.05) However, they were still lower than control group (Fig. 4A, B, C).

The ROT group showed a significant increase in the level of striatal acetylcholine than the control group (72.91 ± 2.28 vs. 32.08 ± 2.25 U/ml, respectively; *P* ≤ 0.05). However, ROT + MaR1 group showed a significant decrease in striatal acetylcholine compared to ROT group (46.75 ± 0.93 U/ml, respectively; *P* ≤ 0.05). The level of striatal dopamine was still substantially lower, and the level of striatal acetylcholine was substantially higher than control group (Fig. 4D).

#### Oxidative stress results

**Fig. 5:**
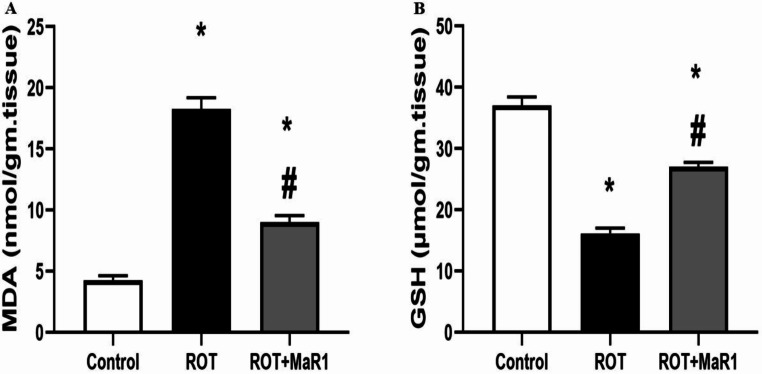
Maresin-1 impact on striatal malondialdehyde (nmol/gr of tissue) (**a**) and reduced glutathione (nmol/gr of tissue) (**b**) in rotenone-induced Parkinson’s disease. Data represented as mean ± SD (*n* = 10) and group comparisons were made utilizing the ANOVA test. ROT + MaR1 (rotenone-induced Parkinson’s disease treated with Maresin-1) and (MDA) malondialdehyde and (GSH) reduced glutathione. *p < 0.05 is significant when compared to the control, and # *p* < 0.05 is significant when linked to the ROT group

In the ROT group, there was a significant elevation in striatal MDA in comparison with control group (18.28 ± 0.92 vs. 4.28 ± 0.35 nmol/gr of tissue, respectively; *P* ≤ 0.05). ROT + MaR1 group exhibited a significant reduction in striatal MDA compared to the ROT group (9.00 ± 0.56 nmol/gr of tissue; *P* ≤ 0.05), while it was still substantially higher than the control group (Fig. 5A).

The ROT group showed a substantial reduction in the striatal GSH level in comparison with control group (16.16 ± 0.85 vs. 37.08 ± 1.35 nmol/gr of tissue; *P* ≤ 0.05). However, ROT + MaR1 group showed a significant increase in striatal GSH compared to the ROT group (27.05 ± 0.71 nmol/gr of tissue; *P* ≤ 0.05). However, its level was still substantially lower than the control group (Fig. 5B).

#### Inflammatory markers results

**Fig. 6:**
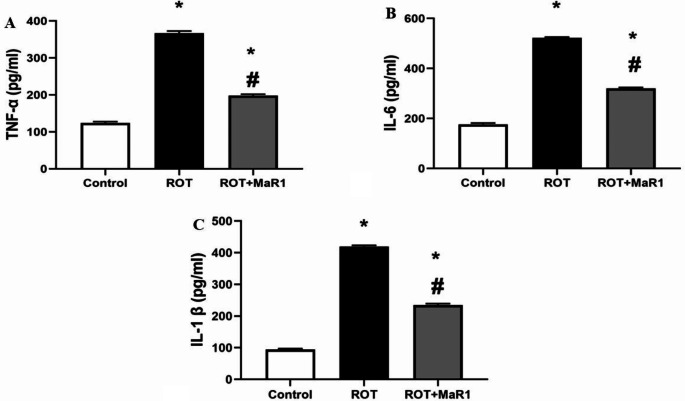
Maresin-1 impact on striatal tumor necrosis factor α (pg/ml) (**a**), interleukin 6 (pg/ml) (**b**), and interleukin 1β (pg/ml) (**c**) in rotenone-induced Parkinson’s disease. Data represented as mean ± SD (*n* = 10) and group comparisons were made utilizing the ANOVA test. ROT (rotenone-induced Parkinson’s disease), ROT + MaR1 (rotenone-induced Parkinson’s disease treated with Maresin-1), TNF-α (tumor necrosis factor α), IL-6 (interleukin 6), and IL-1β (interleukin 1β). *p < 0.05 is significant when compared to the control, and # *p* < 0.05 is significant when linked to the ROT group

In the ROT group, there was a significant increase in striatal TNF-α, IL6, and IL-1β in comparison with the control group (367.33 ± 5.35 vs. 124.16 ± 3.65 pg/ml, 522 ± 3.57 vs. 176.83 ± 4.7 pg/ml, and 419.66 ± 3.32 vs. 94.83 ± 2.31 pg/ml, respectively; *P* ≤ 0.05). However, ROT + MaR1 group exhibited a significant reduction in striatal TNF-α, IL6, and IL-1β compared to the ROT group (198.33 ± 3.44 pg/ml, 320.5 ± 3.93 pg/ml, and 235.33 ± 4.13 pg/ml, respectively; *P* ≤ 0.05). However, their levels were still substantially higher than the control group (Fig. 6).

### Striatal JAK1 and STAT3 genes expression results

**Fig. 7:**
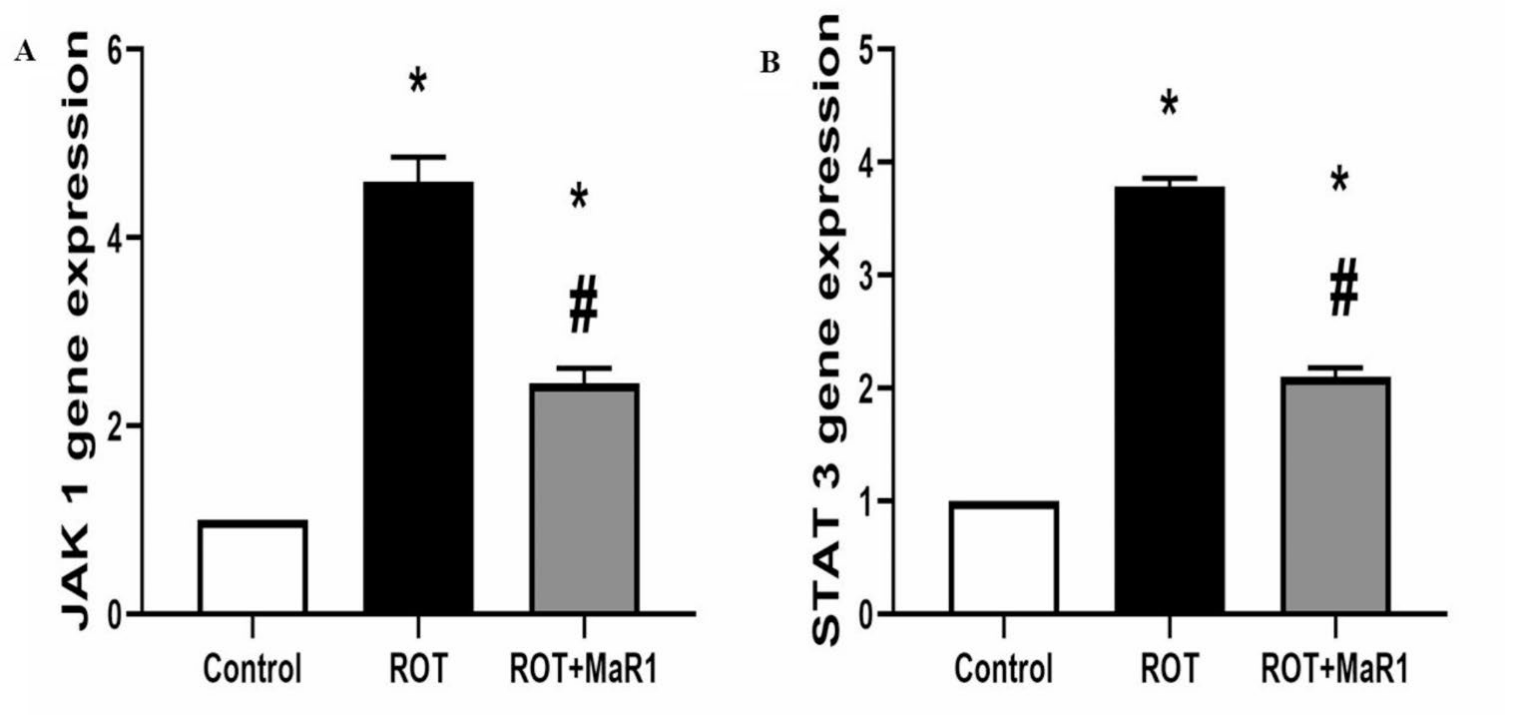
Maresin-1 impact on striatal JAK1 (**a**) and STAT3 (**b**) gene expression in rotenone-induced Parkinson’s disease. Data represented as mean ± SD (*n* = 10) and group comparisons were made utilizing the ANOVA test. ROT (rotenone-induced Parkinson’s disease), ROT + MaR1 (rotenone-induced Parkinson’s disease treated with Maresin-1), JAK (the Janus kinase), and STAT (signal transducers and activator of transcription signaling pathway)

A significant increase in the ROT group gene expression of striatal JAK1 and STAT3 genes (4.6 ± 0.26, and 3.79 ± 0.07, respectively; *P* ≤ 0.05) was observed in comparison with control group. However, ROT + MaR1 group showed a significant reduction in the gene expression of JAK1 and STAT3 genes compared to the ROT group (2.45 ± 0.16 and 2.1 ± 0.08, respectively; *P* ≤ 0.05). However, their levels were still substantially higher than the control group (Fig. 7).

*p < 0.05 is significant when compared to the control, and # *p* < 0.05 is significant when linked to the ROT group.

### Histological Results

**Fig. 8:**
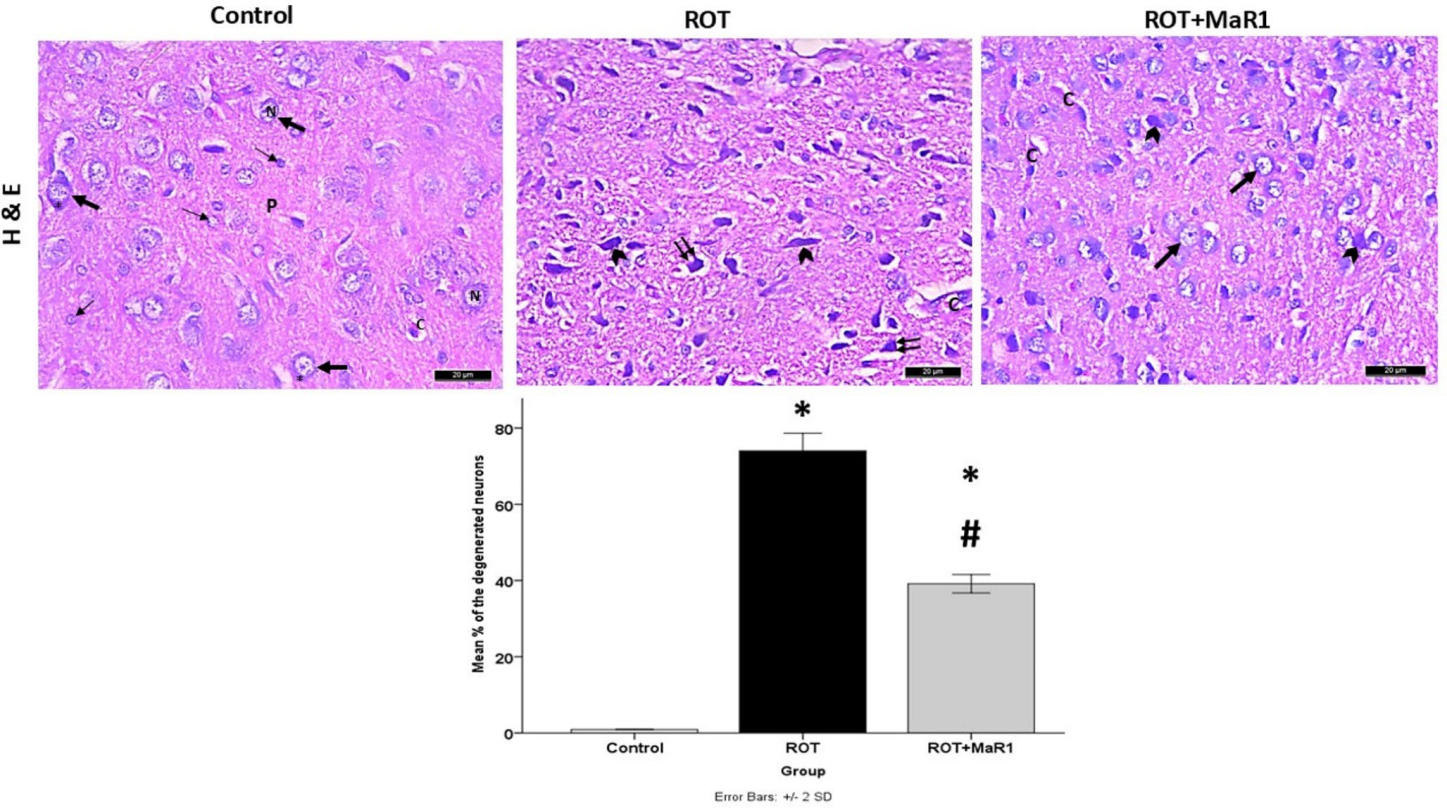
Representative photomicrographs of the H&E-stained sections in the substantia nigra pars compacta of the different experimental groups. The control group showed variable sizes and shapes of neuronal perikarya (thick arrow) with large pale vesicular nuclei (N) and basophilic cytoplasm (asterisk). Some glial cells with small nuclei could be observed (thin arrow). All are embedded within the neuropil (P). A normal blood capillary (C) is noted. ROT group showing degenerated cells with condensed darkly stained pyknotic nuclei (arrowhead). Some have perinuclear halos (double arrow). A dilated blood capillary (C) is noticed. ROT + MaR1 group exhibiting normal neurons (thick arrow). However, some shrunken darkly stained neurons with condensed darkly stained pyknotic nuclei (arrowhead) are still present. Blood capillaries (C) could be observed. The chart demonstrates the % of the degenerated neurons. Data represented as mean ± SD (*n* = 10) and group comparisons were made utilizing the ANOVA test. *p < 0.05 is significant when compared to the control, and # *p* < 0.05 is significant when linked to the ROT group (H&E x400, scale bar = 20 μm)

The brain of control rats revealed normal structures of the substantia nigra compacta (SNc). Variable size and shape of neural perikaryal with vesicular nuclei and basophilic cytoplasm were observed. Normal glial cells and blood capillaries were also observed embedded within the neuropil. The majority of the ROT group’s SNc neurons showed remarkable signs of degeneration, including highly discolored, smaller nuclei with perinuclear halos. Dilated blood capillaries were also observed. On the other hand, the administration of MaR1 ameliorated most of the degenerative changes in the SNc generated by ROT. Most of the neurons in this group had normal structures. However, some had shrunken deeply stained nuclei. A substantial increase in the % of the degenerated neurons of the SNc in the ROT group in comparison with control (74.04 ± 2.30 vs. 0.89 ± 0.04; *P* < 0.001) was observed. The ROT + MaR1 group revealed a substantial reduction in the % of degenerated neurons compared to the ROT group (39.17 ± 1.21 vs. 74.04 ± 2.30; *P* < 0.001). However, a substantial difference between the ROT + MaR1 group and the control one was still present (*P* < 0.001) (Fig. 8).

### Immunohistochemical Results

#### Caspase-3 Immunoreaction

**Fig. 9:**
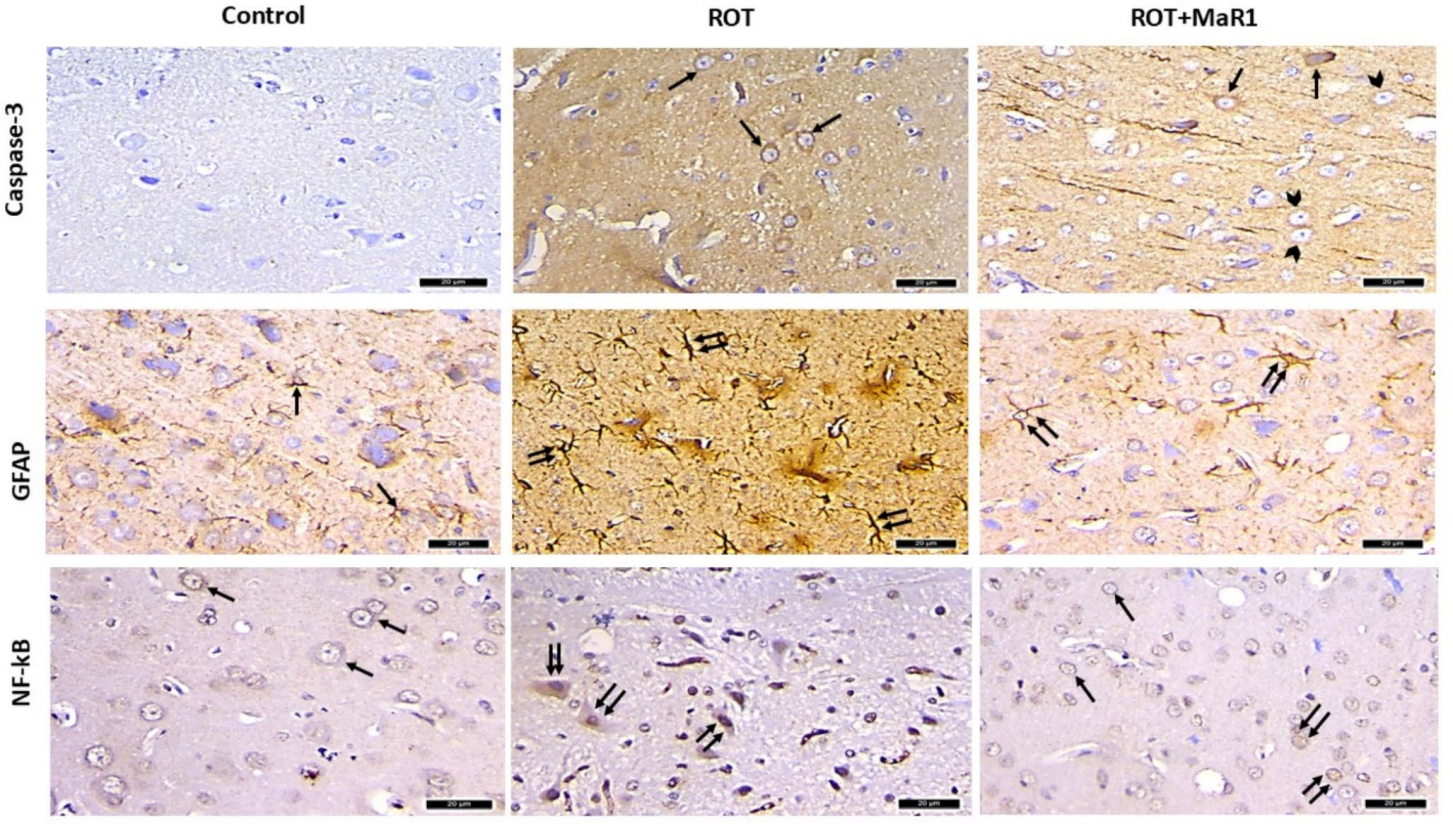
Representative photomicrographs of the immunostained sections in substantia nigra pars compacta of the different experimental groups. Caspase-3 immunostained sections exhibited positive immunoreaction (arrow) in the cytosol of most of the neurons in the ROT group. Meanwhile, in the ROT + MaR1 group, some neurons are positively stained (arrow) and others are negatively stained (arrowhead). GFAP immunostained sections showing few small astrocytes (arrow) of the control. The ROT group showed numerous large astrocytes (double arrow). The ROT + MaR1 group showed few large astrocytes (double arrow). NF-kB immunostained sections exhibited positive cytoplasmic expression (arrow) in the neurons of the control group and positive nuclear immunoreaction (double arrow) in most of the neurons of the ROT group. The ROT + MaR1 group showed cytoplasmic expression of the NF-kB immunoreaction (arrow) in most of the neurons while some neurons have positive nuclear immunoreaction (double arrow) (immunohistochemical staining x 400, scale bar = 20 μm)

**Fig. 10:**
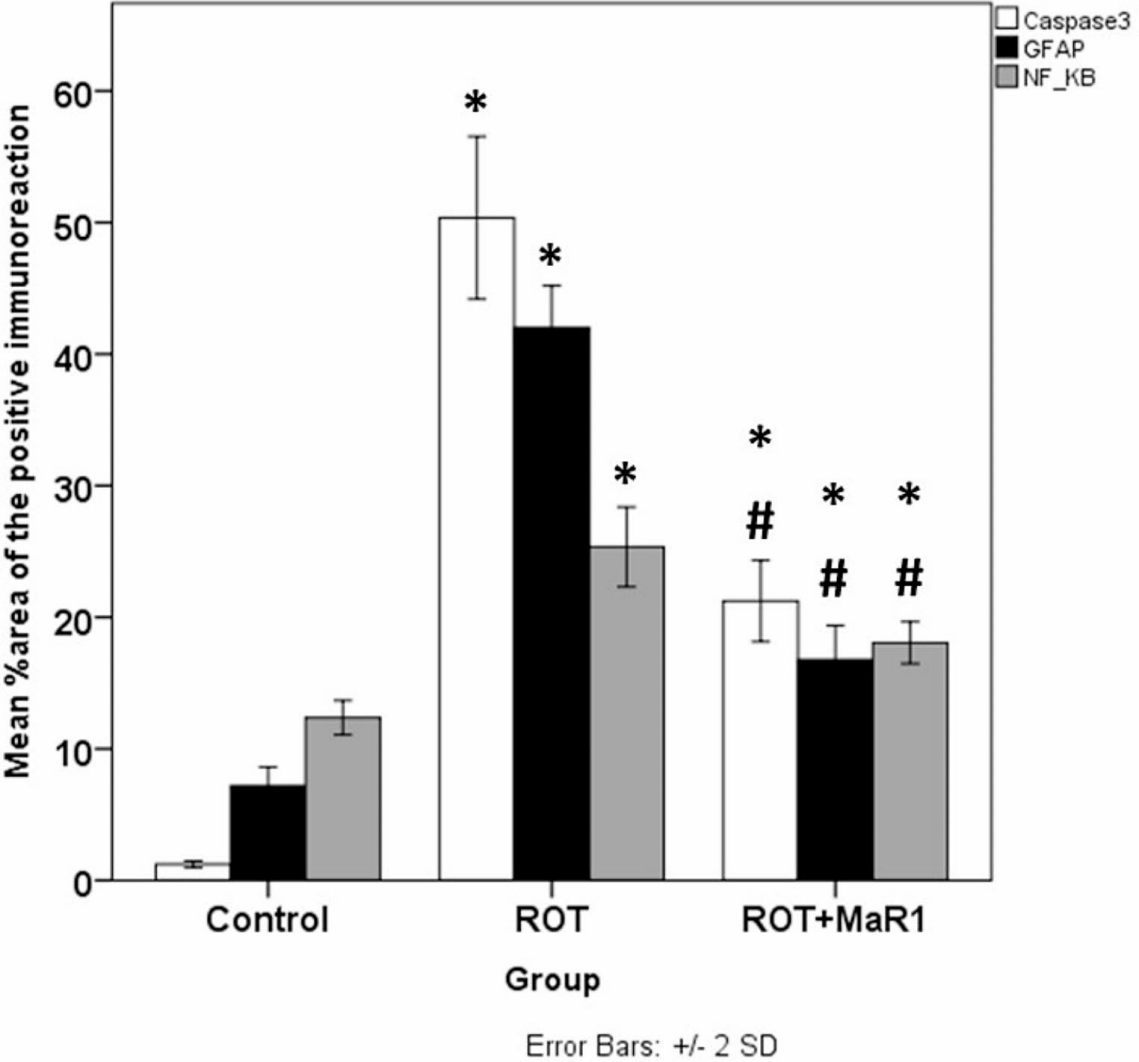
A chart demonstrating the % area of caspase-3, GFAP, and NF-kB positive immunoreaction. *Data represented as mean ± SD (*n* = 10) and group comparisons were made utilizing the ANOVA test. ROT (rotenone-induced Parkinson’s disease) and ROT + MaR1 (rotenone-induced Parkinson’s disease treated with Maresin-1), *p < 0.05 is significant when compared to the control, and # *p* < 0.05 is significant when linked to the ROT group

A substantial elevation in the % area of caspase-3 positive immunoreaction was observed in the ROT group compared to the control (50.23 ± 3.09 vs. 1.21 ± 0.13; *P* < 0.05). On the contrary, the ROT + MaR1 group exhibited a substantial downregulation in the caspase-3 immunopositive cells in comparison with the ROT group (21.22 ± 1.54 vs. 50.35 ± 3.09; *P* < 0.05). However, a substantial difference between the control and ROT + MaR1 was still reported (*P < 0.05*) (Figs. 9 and 10).

#### GFAP Immunoreaction

A substantial upregulation in the % area of the GFAP immunopositive reaction was noted in the ROT group in comparison with the control (41.99 ± 1.60 vs. 7.18 ± 0.72; *P* < 0.001). The ROT + MaR1 revealed a substantial downregulation in the GFAP-positive immunoreaction compared to the ROT group (16.77 ± 1.29 vs. 41.99 ± 1.600; *P* < 0.001). However, there was a substantial difference between ROT + MaR1 and control groups (*P < 0.001*) (Figs. 9 and 10).

#### NF-kB Immunoreaction

The % area of the NF-kB positive immunoreaction was substantially increased in the ROT group compared to the control (25.34 ± 1.51 vs. 12.36 ± 0.65; *P* < 0.001). On the contrary, a significant downregulation of the NF-kB positive immunoreaction was noted in the ROT + MaR1 group in comparison with the ROT group (18.06 ± 0.79 vs. 25.34 ± 1.51; *P* < 0.05). A substantial difference between the control and ROT + MaR1 groups was still reported (*P < 0.05*). The NF-kB positive immunoreaction in the control group showed mostly cytoplasmic expression. However, most of the neurons in the ROT group exhibited positive nuclear expression of the NF-kB immunoreaction. On the other hand, the ROT + MaR1 group revealed mainly cytoplasmic expression with some neurons with positive nuclear immunoreaction (Figs. 9 and 10).

## Discussion

We have emphasized the beneficial impacts of MaR1 against behavioral and motor activity impairments, oxidative stress, neuroinflammation, immunoreaction, and histopathological changes caused by rotenone in rats. Rotenone supplementation is one of the best models for simulating the neuropathological and behavioral characteristics of PD. Currently, rats’ subcutaneous injection of rotenone is a convenient, effective, and commonly utilized method for the induction of PD symptoms [23]. Epidemiologic research has connected exposure to environmental toxins with mitochondrial toxicity with selective vulnerability to nigrostriatal degeneration and a higher risk of PD. Rotenone, a common pesticide, selectively inhibits the electron transport chain’s mitochondrial complex I. Rotenone is a potent inflammatory and oxidative damaging agent that can generate PD-like symptoms in rats [5, 24].

We did not incorporate a group in which only MaR1 was administered as in previous study, there was no significant difference between control group and MaR1 treated group regarding inflammatory cytokines and NF-κB [25]. Also, in a mouse model of perioperative neurocognitive disorders, there was no significant difference between control group and MaR1 treated group on brain [26].

In the current study, rotenone administration induced a downfall in the motor behavioral pattern in rats manifested as reduced period spent on the rotarod, reduced grip strength test response, reduced locomotor performance in open field test, and reduced stepping test performance, in addition to increased striatal acetylcholine levels and a manifest reduction in striatal dopamine. This proves the induction similar to that observed in idiopathic disease. This is aligned with previous studies indicating that rotenone injection for 5 weeks deteriorated behavioral aspects with a reduction in exploration and locomotion, impaired motor coordination, cognitive dysfunction, reduced striatal dopamine, and enhanced acetylcholinesterase activity level [27–32]. The rotarod test is widely utilized for assessing mouse PD model motor skills. It is impacted by several factors, including stiffness, bradykinesia, and a lack of coordination [3].

These results were consistent with histopathological findings revealing the presence of marked neuronal degeneration and apoptotic changes in the SN. These apoptotic changes were confirmed by the upregulation of caspase-3 immunoreaction in the ROT group in comparison with control. These results agree with the findings of previous studies [33–35]. In agreement with these results, Yuan et al. [36] reported an increase in caspase-3 levels in the SN regions of ROT rats. referring to the presence of abnormal α-synuclein aggregation in the dopaminergic neurons which results in the increased expression of caspase-3.

The present study exhibited amelioration of most motor behavioral and histopathological changes in the ROT + MaR1 group compared to the ROT group indicating that MaR1 can protect against neurodegenerative changes in experimentally induced PD. The neuroprotective effect of MaR1 was in accordance with Yin et al. who stated that MaR1 played a neuroprotective role in a mouse model of Alzheimer’s disease (AD) [37]. Moreover, Wei et al. observed that MaR1 administration in mice after sciatic nerve crush injury promoted injured nerve regeneration [38]. Also, Mar1 exhibited neuroprotection in postoperative cognitive dysfunction [39], Sepsis-associated encephalopathy [40]. MaR1 was found to have a protective effect, not only on nervous tissues but also on other tissues as in experimental models of acute kidney injury and chronic hepatic damage [41, 42]. In the present study, the SN neurons of rats from the ROT + MaR1 group also exhibited a remarkable downregulation of the caspase-3 immunoreactivity in comparison with the ROT group. This was in agreement with the findings of Tang et al. [43].

Furthermore, we observed a substantial upregulation in GFAP in the ROT group that was significantly reversed in ROT + MaR1 rats. This was consistent with the findings of Murakami et al. [44]. GFAP is a major cytoskeletal protein in astrocytes. It functions as an inflammatory marker produced from astrocytes and an indication of reactive astrogliosis. GFAP expressions are significantly elevated in neurodegenerative diseases, including AD and PD [45]. Both the striatum and the globus pallidus showed increased GFAP immunostaining intensity in response to experimental parkinsonism and PD [46]. By generating neurotrophic chemicals, and antioxidants, and removing neural waste products such as aggregated α-synuclein and damaged mitochondria, astrocytes safeguard neurons. On the other hand, under pathological circumstances, activated microglia cause astrocytes to release neurotoxic substances causing neuronal damage and neurodegeneration and initiating an inflammatory response [47]. Astrocyte dysfunction results in the transmission of α-synuclein from neurons to astrocytes [48]. Intracellular α-synuclein inclusions in astrocytes lead to an inflammatory response and affect the essential roles of these cells resulting in dopaminergic neuronal loss as clarified by Gu et al. [49].

GDNF is a powerful neurotrophic factor for dopaminergic neurons in the midbrain secreted by astrocytes. Recombinant human GDNF improves the survival and restoration of the injured nigrostriatal dopamine system and promotes motor functions and dopamine uptake in rat models of PD [50]. Reduced levels of GDNF were observed in PD patients in comparison with control which was suggested to be the primary risk factor for PD [51–53]. Reduced GDNF was observed in ROT rats suggesting that deficit in GDNF expression becomes increasingly important for dopaminergic dynamics and associated behaviors. On the supplementation of MaR1 to ROT rats, striatal GDNF level was increased significantly.

Oxidative stress is among the factors linked to PD. Elevated generation of reactive species of oxygen (ROS) during oxidative stress causes a discrepancy between the body’s antioxidant and oxidative capacities, which were reflected in the current study by the measurement of the MDA and GSH levels. Rotenone elevated MDA and reduced GSH (an endogenous antioxidant). This was consistent with earlier studies [54] indicating that rotenone significantly increases brain oxidative injury, as evidenced by an increase in MDA and reduced oxidative defense system. Since rotenone interferes with mitochondrial complex I and upsets the antioxidant system, it causes the buildup of ROS. Since neurons demand a lot of energy and have a high lipid content, they are susceptible to oxidative stress. When MDA combines with proteins and DNA bases, it produces genotoxic lipid peroxidation products causing neuroinflammation and ultimately cell death [55]. While systemic rotenone infusion consistently inhibits the brain’s mitochondrial complex I, rotenone treatment selectively degenerates the nigrostriatal dopaminergic pathway [56]. The morphology and function of astrocytes in the brain can be impacted by oxidative stress. Free radicals trigger multiple inflammatory related signaling pathways in astrocytes and promote the production of inflammatory factors [57]. MaR1 supplementation resulted in a substantial decline in MDA and an increase in GSH. MaR1 protective effect may be due to the inhibition of ROT-induced mitochondrial impairment and decreased production of ROS. This coincides with the findings of Gu et al. who reported that MaR1 alleviated lung injury in mice, caused less inflammatory cytokine release, and increased survival rate via reducing mitochondrial dysfunction [58–60].

In the current study, rotenone treatment had a detrimental effect on striatal TNF-α, IL-6, and IL-1β. Neurodegeneration may be influenced by inflammation. According to research, PD patients have higher than normal amounts of inflammatory markers. Proinflammatory cytokine activation causes neuroinflammation, upregulates the JAK/STAT signaling pathway, and activates other transcription factors including NF-κB. This, in turn, results in microglial activation and dopaminergic neuron autophagy, both of which are factors in PD [61]. To investigate MaR1’s downstream effects, we measured NF-kB, JAK1, and STAT3.

The JAK/STAT pathway is the main signaling pathway used by cytokines. In addition to many other biological processes such as immunity, proliferation, differentiation, apoptosis, inflammatory reactions, and oncogenesis, it aids in intracellular signal transmission. Conditions like cancer, inflammation, and neurological deficits are caused by the dysregulation or phosphorylation of the JAK/STAT pathway’s components [7]. Quantitative PCR revealed the upregulated mRNA expression of JAK1 and STAT3 in ROT rats. Activation of the JAK/STAT pathway can upregulate numerous cytokine receptors including TNF-α, IL-1β, and IL-6 which can further activate JAK that can augment the inflammatory response. By preventing microglial activation and the release of proinflammatory cytokines and chemokines, suppressing the JAK/STAT pathway can stop neuroinflammation and neurodegeneration [9, 62].

A family of transcription factors known as NF-κB controls genes related to inflammation, immunology, and cell survival [63]. Because NF-κB is covalently bonded to its inhibitor protein, IκB, it is contained in the cytoplasm of unstimulated cells. Activation of NF-κB and its translocation to the nucleus occurs when cells are exposed to various stimuli, such as bacterial endotoxins, oxidative stress, and the inflammatory cytokines, TNF-α and IL-1β. The cellular response to oxidants or inflammatory reactions is associated with activated NF-κB, which binds to target genes, such as those of many proinflammatory mediators [64]. A substantial increase was observed in the NF-κB immunoreactivity in the SN of ROT rats. Our results were in agreement with those of Yuan et al. who found upregulation of the endogenous nuclear p65 subunit of NF-κB in the SN lesioned by rotenone [63, 65]. These findings may indicate the role of the NF-κB inflammatory signaling pathway in the development of PD-induced neurodegeneration. The activation of NF-κB promotes the transcription of NOD-like receptor protein 3 (NLRP3). It is well known that microglia use NLRP3 to identify danger signals resulting in the formation and activation of the NLRP3 inflammasome which contributes to the progression of neurodegeneration with the activation of caspase-1 and IL-1 as clarified by Li et al. and Xu et al. [32, 66].

We assessed MaR1’s impact on the activation of the JAK/STAT pathway in rotenone-induced PD. MaR1 significantly suppressed the JAK/STAT activation. MaR1 could also inhibit the JAK/STAT pathway to protect against spinal cord injury [15], sepsis-induced lung injury [62], and developmental neurotoxicity [67]. In the present study, some neurons from the substantia nigra of rats from the ROT + MaR1 group exhibited a remarkable downregulation of the NF-κB. Few neurons showed lower NF-κB immunoreactivity in their nuclei in comparison with the ROT group, while other neurons showed NF-κB immunoreactivity only in the cytoplasm. From these findings, we can assume that MaR1 can perform its neuroprotective effect against rotenone through the reduced activation of NF-κB. MaR1 could also reduce the NF-κB activation in in vitro cell models of AD neuroinflammation, a model of hyperalgesia, and a rat model of chronic hepatic damage [42, 68, 69].

The protective function of MaR1 in this investigation may also be related to its anti-inflammatory properties and its ability to prevent the activation of microglia and astrocytes. This is confirmed by the significant downregulation of the GFAP immunoreactivity in the ROT + MaR1 group compared to the ROT group. In line with this explanation, Fattori et al. observed that MaR1 treatment in a mouse model of hyperalgesia reduced the production of proinflammatory cytokines (TNF-α and IL-1β), the microglia activation, and GFAP in the spinal cord of these mice [68]. Similarly, Yin et al. reported that MaR1 could attenuate inflammation in a mouse model of AD by inhibiting proinflammatory activation of microglia and astrocytes. They added that MaR1 could also increase the expression of anti-inflammatory cytokines such as IL-10 and IL-2 [37, 70]. The study’s results provide strong support for past research suggesting that MaR1 may be a different kind of treatment to mitigate the drawbacks of the anti-inflammatory drugs now on the market [71].

The treated groups did not fully normalize all parameters, although they showed significant improvements compared to the ROT group. This suggests the need for further research on the effects of Mar1 in Rot-induced PD, particularly with varying doses to identify the most effective dosage for optimal improvement. We will address this issue in the future.

## Conclusion

Based on the findings reported in the current study, we offer an evaluation of a potential route by which rotenone could function during PD. Furthermore, our data declare a protective effect of Maresin-1 against rotenone-induced PD and reveal that the protection mechanism involves suppressing inflammation and apoptosis, and antioxidant effects. Moreover, the implication of the JAK/STAT pathway within the MaR1 + ROT rats provides a viable readout to compare the potency and duration of action of the test compound.

## Data Availability

The data are available on request from the corresponding author.
